# A Motivational Model of BCI-Controlled Heuristic Search

**DOI:** 10.3390/brainsci8090166

**Published:** 2018-08-31

**Authors:** Marc Cavazza

**Affiliations:** Department of Computing and Information Systems, University of Greenwich, London SE10 9LS, UK; m.cavazza@gre.ac.uk; Tel.: +44-20-8331-8512; Fax: +44-20-8331-8665

**Keywords:** augmented cognition, brain–computer interfaces, superintelligence, heuristic search

## Abstract

Several researchers have proposed a new application for human augmentation, which is to provide human supervision to autonomous artificial intelligence (AI) systems. In this paper, we introduce a framework to implement this proposal, which consists of using Brain–Computer Interfaces (BCI) to influence AI computation via some of their core algorithmic components, such as heuristic search. Our framework is based on a joint analysis of philosophical proposals characterising the behaviour of autonomous AI systems and recent research in cognitive neuroscience that support the design of appropriate BCI. Our framework is defined as a motivational approach, which, on the AI side, influences the shape of the solution produced by heuristic search using a BCI motivational signal reflecting the user’s disposition towards the anticipated result. The actual mapping is based on a measure of prefrontal asymmetry, which is translated into a non-admissible variant of the heuristic function. Finally, we discuss results from a proof-of-concept experiment using functional near-infrared spectroscopy (fNIRS) to capture prefrontal asymmetry and control the progression of AI computation of traditional heuristic search problems.

## 1. Introduction and Rationale

Human augmentation aims at extending human cognitive abilities, often in a situated, task-specific fashion. Previous research has demonstrated through various implemented prototypes and experiments the feasibility of extending human perceptive abilities or information processing and decision-making abilities [1,2]. In the latter case, Artificial Intelligence (AI) techniques are poised to play a significant role in providing the task-specific information processing power supporting the augmentation aspects. A constant feature, and a defining aspect of human augmentation, is that locus of control remains strictly with the human, and the human task dynamics is left largely unchanged. The information processing ability provided by the augmenting system is inserted into the natural human activity, in a user-centred way, largely like augmented reality systems enhance world perception through advanced imaging abilities. One such example is cortically coupled perception [3], in which user active analysis of satellite images is augmented by the EEG-based detection of perceptive signals: in this experimental system, the human analyst approach to image exploration is essentially unchanged. 

Although human augmentation systems have been developed prior to the popularisation of Brain–Computer Interfaces (BCI), these have taken a more prominent role in recent years, as they offer a seamless mechanism to capture elements of human cognitive processes in a way that enables the synchronisation of computations [1,2]. With the rise of autonomous intelligent systems, a new application of human augmentation has been suggested in order to keep humans in control of autonomous AI systems whose performance could potentially exceed even that of human experts.

After years of inflated expectations about AI, recent progress, primarily in machine learning, has led to much-advertised successes [4,5] and renewed confidence in AI advances. Paradoxically, this situation has also fuelled the preoccupation that AI progress will eventually constitute a threat to the well-being of humans. Researchers across a variety of disciplines have taken the stage to forewarn of the potential adverse consequences of unregulated AI progress, amongst which the automation of white-collar jobs [6], the development of AI-endowed autonomous warfare [7], or even the rise of superintelligent AI entities [8,9]. Whether or not this superintelligence threat will materialise, the shorter-term availability of advanced AI systems able to outperform human experts at an increasing number of professional tasks is sufficient to justify research into hybrid cognitive systems. 

The imbalance between humans and AI systems stems largely from the inability of humans to engage with, even less control, the automatic reasoning mechanisms underpinning AI systems. This stems largely from the scale and pace of data processing, which is not compatible with the timeline of human decision making. It can also be noted that this lack of surveyability is not strictly attributable to a representational issue (e.g., sub-symbolic versus symbolic), as complex search systems, including statistical ones such as Monte Carlo tree search [5], remain based on the discrete step granularity of search. 

There is thus a case for additional research exploring a synergy between humans and AI systems, which should aim at endowing humans with high-level control abilities sufficient to steer the flow of AI computation, irrespective of its low-level details, while preserving an understanding of the computation goal. Several authors have specifically suggested human augmentation as a potential solution to the threat posed by superintelligence, augmentation being often achieved through BCI implementations. Although most of these proposals remain largely underspecified, and some are not always consistent with the state-of-the-art of BCI systems, it is worth briefly reviewing the commonalities between them. Bostrom dedicates a section of his book [9] (p. 169) to the potential of BCI for controlling superintelligence: however, his analysis is moderately optimistic, largely because he equates BCI with its invasive implementations (depth electrodes or electrocorticography (ECoG)), and raises legitimate concerns about acceptance, maturity of the technology, side effects, and user safety. Kennedy [10] suggests BCI-based augmentation primarily as an alternative pathway to autonomous superintelligence rather than as a control mechanism, and rightly identifies BCI signal/information bandwidth as a major challenge. Skulimowski reviews several candidate scenarios for superintelligence [11], one of which involves human control through BCI connection. Finally, Barrett and Baum, in their review of pathways to (artificial) superintelligence [12], discuss several risk reduction interventions, one of which includes human augmentation through BCI. 

Despite being initially identified as human augmentation, it would actually imply a paradigm shift, because the main information processing cycle would be driven by the autonomous AI rather than by the human, as is customary in traditional cognitive augmentation systems; here, the human user would be included in a supervisory capacity. To be successfully implemented, this framework should not require a transformation of the AI technology to support user intervention (e.g., mixed initiative), as it might compromise efficiency and the very advantage of autonomy. The challenge we are addressing here consists precisely in providing minimally invasive supervision by the human user. To summarise previous literature, the rationale for providing supervisory control can be described from two complementary perspectives: (i) controlling the nature of the solution during its calculation (in terms of optimality, solution parameters, or other application-related criteria), and (ii) ensuring compliance with ethical standards. 

In this paper, we introduce a candidate framework aimed at controlling the behaviour of autonomous AI systems using a BCI. This unique combination of BCI and AI is meant to integrate BCI input directly at the level of AI algorithmic computation so as to influence inner mechanisms in a principled manner, being compatible with typical BCI information bandwidth and without imposing additional restrictions on the nature of AI computation. 

Although the current approach shares important aspects with augmented cognition, it differs fundamentally by the fact that the main computation is actually determined by autonomous AI mechanisms with the user supervising the computation rather than actually driving the task, as in cortically coupled vision or enhanced information retrieval [2]. The combination of the user and the AI system forms a hybrid cognitive system in which some high-level executive control would be retained by the human, while autonomous AI would form the main cognitive process. To account for the complex spectrum of human–system integration, previous literature has used terminology such as symbiotic systems [13] or human–computer confluence [14], and we should refer to our approach as BCI-controlled heuristic search, categorising the type of system we are aiming for as a hybrid cognitive system. 

One of the main objectives is to achieve consistency between user intentions and the principles that can affect the progression of AI computation: to that effect, we will review several principles that reconcile active BCI, user cognitive mechanisms, and AI computation dynamics. In the next sections, after introducing the issues emerging from autonomous AI systems and reviewing relevant BCI augmentation systems, we discuss basic AI mechanisms (i.e., heuristic search) that can serve as a target for BCI influence. We then explore cognitive processes that could be harnessed to provide control over AI computation. We will emphasise cognitive mechanisms around motivation, which range from reward expectation to risk propensity, trying to relate them to compatible concepts that characterise the progress of AI computations in terms of result anticipation. Even before being fully fledged from a theoretical perspective, this framework has been the object of early proof-of-concept testing through a fully implemented prototype, whose results are briefly analysed as additional input into the proposed framework. Finally, we take a system design perspective to review the conditions for a successful implementation of the framework, as well as possible implementation variants. 

## 2. A Motivational Model of AI Control

Theoretical research on superintelligence has suggested various approaches and mechanisms to ensure it will stay under human control. In the first instance, we will consider that, from a technical perspective, the human augmentation mechanisms proposed for superintelligent systems should not fundamentally differ from those to be associated with shorter-term autonomous AI systems endowed with advanced planning, decision making, or information analysis abilities. Bostrom has advocated one specific control mechanism, which he characterises as motivation selection methods [9] (p. 169), or methods that would shape the nature of the solution produced by the AI system. While his original discussion is influenced by a rather anthropomorphic view of the AI’s goals and intentions, this philosophy can be extended to more technical visions of AI systems to describe the type of solution produced, whether this type is defined in terms of goal properties or solution properties (when the shape of the solution, seen as a sequence of actions towards the goal, constitutes a desirable property of the output). For instance, instead of indirect normativity [9] (p. 169) influencing the set of values used by the AI in the pursuit of a solution, the nature of a solution could be shaped by the user according to shared concepts characterising the nature of the solution. Candidate concepts would include reward anticipation, risk taking, and solution optimality: we shall develop in the forthcoming sections how these concepts can be related to cognitive motivational dimensions and how they can be made accessible to BCI input. In the next section, we will first lay out some AI basic mechanisms that rest at the heart of many AI systems and can constitute a target for the user-based influence of AI computation. 

In the above model, motivation has been defined primarily in relation to goal setting and goal pursuit. Recent research in cognitive neuroscience [15] uses a compatible definition of motivation that can be made interoperable with AI technology concepts. In addition, it identifies the involvement of specific brain regions in a way that supports the design of appropriate BCI. From a cognitive perspective as well, motivation is conceived of as being goal directed [16,17]. The relationship to the goal has been further refined into planning and implementing stages [18], also suggesting that goal setting is primarily motivational, while goal striving is best characterised in terms of volitional factors [18]. According to [16], the neural systems implicated in the internal representation of cognitive goals overlap significantly with those dealing with the generation of motivated behaviours. In particular, the lateral prefrontal cortex (PFC) might serve as a convergence zone in which motivational and cognitive variables are integrated [16]. 

The identification of specific brain regions whose activation may reflect motivational dimensions is an essential step in designing appropriate BCI. In terms of activity measurement, there is a substantial body of work associating PFC asymmetry with motivational direction [19], which originates with the study of approach/withdrawal as a motivational dimension [20]. This research has pioneered the measurement of prefrontal asymmetry using EEG signals [20], left asymmetry being associated with the expression of approach. 

The relationship of frontal EEG asymmetry with motivational variables has been recently reviewed by Smith et al. [21] and Harmon-Jones and Gable [17], who have related resting left prefrontal asymmetry to individual differences in self-reported trait approach motivation. In addition, they have found this relationship to be stronger in the context of incentive anticipation. Moreover, there are strong relations between motivation and reward anticipation: for instance, lateral PFC activation is modulated by the level of reward offered [22,23]. Amodio et al. [24] have analysed the correlates of PFC asymmetry from a regulatory perspective. More specifically, they found approach regulation to be most relevant to “pre-goal states”, during which efforts are mobilised towards the goal. This needs to be reanalysed from the prism of a hybrid cognitive system, which could involve a mix of goal setting and goal pursuit depending on the information visible to the user from the AI computation but, in any case, is compatible with a mediation from prefrontal asymmetry.

Prefrontal asymmetry as a marker of approach [17] has been extensively studied by EEG under three different conditions: (i) at rest, (ii) as a dynamic response to a cognitive situation or an affective stimulus, and (iii) under volitional control through neurofeedback (NF). To understand the dynamics of prefrontal asymmetry, it is worth noting that its value is determined approximately for half by its resting value (trait) and for another half by its dynamic value (state): this is in particular what makes it amenable to volitional control through NF, although the trait component may introduce ceiling effects rendering some subjects more prone to dynamic changes than others. 

Functional Magnetic Resonance Imaging (fMRI) studies have been carried out to uncover the anatomical basis of prefrontal asymmetry in the context of motivational phenomena [25,26]. In addition, real-time-fMRI (rt-fMRI) experiments have demonstrated the controllability of prefrontal asymmetry [27] including comparisons to EEG-based NF. Functional Near Infrared Spectroscopy (fNIRS) studies of the PFC have been dedicated to affective interaction [28] as well as executive control. fNIRS is also amenable to NF implementation that supports BCI, and we have successfully used it for BCI-based prefrontal symmetry in a context of distinguishing approach from valence [29]. Harmon-Jones et al. [19] have questioned the exclusive role of the dorsolateral prefrontal cortex (DLPFC) in accounting for BCI signals for approach, on the basis that EEG and metabolic methods, such as fMRI, measure different activities for different cellular populations [30], while noting that EEG findings were still corroborated by experiments with transcranial stimulation (see for instance, [31]). In their most recent review, Harmon-Jones and Gable [17] have considered that fMRI may actually show more complex patterns of activation without this invalidating the central role of DLPFC and the use of EEG to measure prefrontal asymmetry. 

When placing the human user in a position of high-level arbitration of autonomous AI computations, it is tempting to resort to a metaphor of executive control, even more so when resorting to neural signals originating in the prefrontal cortex. A hybrid model of executive control could be envisioned, by redefining executive control for a hybrid cognitive system comprised of the human and the autonomous AI, in which human executive control would apply to the deliberative AI part instead of the human part. One specific question arising when considering cognitive control in the context of hybrid cognitive systems is the extent to which prefrontal cognitive control mechanisms that have been described to operate on internal cognitive mechanisms would apply to hybrid control situations where the generation of hypotheses, or anticipation of rewards, is actually not the result of the human cognitive processes but of their appraisal of the AI calculation progress. 

Smith et al. [21] have actually related EEG prefrontal asymmetry not just to motivation but also executive functions. Current integrative models of executive function control [32] distinguish between hot (affective) and cold (deliberative) executive control and tend to associate the DLPFC with cold control and the orbitofrontal cortex (OFC) with motivation and reward anticipation. This would be consistent with source-localisation studies, which have suggested that frontal EEG asymmetry at rest is mediated by left DLPFC and OFC activation [33].

Yet, Auperle et al. [22] (following, amongst others, [34,35]) have distinguished specific roles for OFC and DLPFC. They suggest that OFC is involved in determining the value of rewards, while DLPFC incorporates these values when planning for the execution of a decision or response. Compatible findings had been reported by Tanaka et al. [36], with OFC involved in learning from the present state and DLPFC in learning from predictable future states. The original work from Wallis and Miller [37], based on a primate model, established that OFC encoded the reward value alone, while DLPFC encoded both the reward value and the forthcoming response. Li et al. [38] have also suggested that subjects could use the DLPFC to dynamically adjust outcome responses depending on the usefulness of action-outcome information, implying that they could make use of instructed knowledge rather than simply trial and error outcomes. The role of the left PFC has been described from a hierarchical perspective alongside a rostro-caudal hierarchy as introduced by Coutlee and Huettel [39]. In that context, the DLPFC, whose activity has the prominent role in PFC asymmetry, is considered to be involved in “mid-level abstraction control”, which would be compatible with the goal-oriented role discussed above. 

Despite the overlap between motivational and cognitive factors in the PFC, it is difficult to conclude that hybrid cognitive systems could implement executive control simply by transposing human cognitive mechanisms and dissociating human executive control from other cognitive processes, the latter being substituted with an AI system, without a better understanding of the actual control signals and required information bandwidth. We should then entrust control of the hybrid system to the motivational component, whose signal properties and cognitive activation are better understood, without ruling out that in the context of observing the progression of AI computations, these may still interfere in part with executive functions. While some details of the framework remain to be refined—in particular, the exact balance between goal definition and goal pursuit—the above discussion contains sufficient evidence of the appropriateness of a motivational framework to support the interactive component of a hybrid cognitive system.

### 2.1. Heurisitc Search in AI Control

Implementing cognitive control over AI computation requires the identification of a target computational element, which is generic enough to support one of more classes of AI systems and would not require altering the nature of AI calculations themselves to deploy explicit control mechanisms. One possible target mechanism would consist of the basic elements of AI computation, such as search. The underlying hypothesis is that altering the basic component of heuristic search offers significant leverage on the behaviour of the entire AI computation that derives from it. Of all the algorithmic components underpinning the implementation of AI systems, heuristic search enjoys a central position and also one that has persisted from the early days of AI problem solving to the most recent successes of AI technology.

Heuristic search is in itself a problem-solving technique supporting direct resolution for puzzles such as the Rubik’s cube [40] or spatialised optimisation problems such as the travelling salesman problem or equivalent problems [41]. It has been embedded in a large range of real-world AI applications, from speech recognition to sequence alignment in bioinformatics and many others [42]. However, its real power derives from its incorporation in complex problem-solving techniques supporting more sophisticated knowledge representation, such as search-based planning, which has become the dominant planning technique [43], or question answering systems [44] of the type popularised by IBM’s Watson™. Within these systems, modifications of the basic search mechanisms are potentially able to affect the generation of solutions in the application’s semantic domain without the requirement for domain control knowledge. 

**Figure 1:**
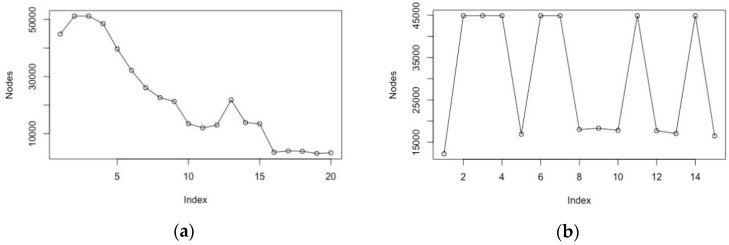
The speed–accuracy trade-off (impact of weighted A* (WA*) on heuristic search performance), illustrated through the reduction in the number of nodes explored to reach a solution. (**a**) Reduction in search space for the 8-puzzle depending on the variation of the weighting coefficient (*x* axis, arbitrary units) (**b**) Restriction of impact depending on the stage of intervention for the 8-puzzle, for a weighting coefficient (0.57) known to reduce the search space.

The most generic mechanism to influence heuristic search is to act upon the heuristic itself: in particular, this mechanism can leverage on search progression in order to influence higher-level AI computations, without requiring ad hoc or application knowledge. The formal properties of heuristic functions have been extensively studied, and as a consequence, the effects of some heuristics’ modification are well understood, and their mathematical properties established. For instance, the behaviour of the entire search process towards an optimal solution is determined by the admissible nature of the heuristic function [45]. It has subsequently been demonstrated that heuristic functions departing from admissibility could be used to trade solution optimality for computational speed (Figure 1). More importantly, it has been established that this departure from optimality could be limited while still showing beneficial effects on search performance: this is referred to as bounded non-admissibility [45] or sometimes ε-admissibility. The main mechanisms to design non-admissible heuristics include dynamic weighting of the heuristic function and focal search. Dynamic weighting consists of allocating different weights to the cost function (g) and the heuristic estimate function (h), generally increasing the latter’s weighting above 0.5. One early implementation of dynamic weighting is Pohl’s depth-dependent dynamic weighting [46], whose rationale is to increase the role of the heuristic component as search progresses towards the goal. This was one of the early demonstrations that heuristics could be modified during the search process itself, such dynamic modifications entailing interactive approaches without requiring the transformation of the baseline search algorithm into a real-time version. The generic approach to dynamic weighting is defined without consideration of search depth simply as a weighted formula for the evaluation function [47], where *n* is the node considered, *g* the cost function, *h* the heuristic estimation function, and ω the weighting coefficient:

*f*(*n*) = (1 − ω) × *g*(*n*) + ω × *h*(*n*). (1)

It has been established that dynamic weighting results in the heuristic not being admissible, such non-admissibility being, however, bounded as a function of the weighting coefficient [48] (which makes dynamic weighting approaches ε-admissible). 

Another major non-admissible search paradigm is known as focal search. It consists of applying a secondary heuristic to refine the selection of the most promising nodes selected by the main heuristic and one of its early descriptions is A*ε [45] (p. 89). The underlying mechanism consists of creating a subset of the most promising nodes under consideration (the OPEN list), this subset being called FOCAL. Instead of selecting the best node from OPEN, the search algorithm will select the best node from FOCAL using the secondary heuristic function to that purpose. The canonical description of FOCAL search has been demonstrated to be ε-admissible, as long as the size of FOCAL is limited, and is actually referred to as A*ε. It should not be confused with methods for combining multiple primary heuristics, which, unlike A*ε, still operate on the original node selection mechanism within the OPEN list. The original description of A*ε made explicit reference to minimising computational costs [45] (p. 89), hence again trading optimality for computational speed, and the secondary heuristic was based on such a computational cost estimate. However, there is no a priori restriction on the nature of the secondary heuristic. In particular, it can be used to incorporate application semantics into the search process, for instance, through the evaluation of specific state configurations. Although these mechanisms have been described as studies of the fundamental properties of heuristic search, they have also generated real-world applications: for instance, a recent implementation of FOCAL search has been used for Unmanned Aerial Vehicles (UAV) coordination through the enhanced conflict-based search mechanism [49]. 

From a hybrid cognitive system perspective, the mechanisms underpinning non-admissibility can constitute appropriate targets for intervention, provided the behaviour of non-admissible variants can be attributed cognitive significance by the user within the motivational framework outlined above. The first illustration of the latter point would be the concept of speed–accuracy trade-off, which is the cornerstone of ε-admissible search [45], and has also been explicitly identified in the cognitive literature on motivation–cognition interaction [50]. 

An alternative cognitive interpretation would be to consider risk as a unifying concept. From the AI perspective, departing from admissibility carries the risk of producing a solution whose cost is higher than that of the optimal solution [51]. From a cognitive neuroscience perspective, this would be based on several findings on the correlation of resting PFC asymmetry with sensation seeking and risk acceptance [52] or the effect of transcranial direct current stimulation-induced right PFC suppression on risk acceptance [31]. 

However, on the AI side, non-admissibility actually corresponds to bounded risks (i.e., somehow acceptable by nature) and, in many cases, deviations from the optimal solution are actually rather minimal. There is no proper quantification of risk in non-admissible heuristic search unlike the case with explicit risk-based approaches to search such as R_δ_* [45]. This algorithm bases node expansion on the error probability distribution for the heuristic function, thereby formalising the risk of ignoring a more promising direction in search than the one taken. Even so, insofar as such risk may not be clearly visible to subjects during interaction, it is unlikely to redefine the motivational framework as a risk-based approach because of the lack of conscious exposure to risk taking. 

The basic mechanism we have chosen for influencing heuristic search progression through bounded non-admissibility consists of dynamic weighting of the A* heuristic function. There is significant background work on dynamic weighting from Pohl’s early work on depth-dependent dynamic weighting [46] to recent anytime variants of A*, in which the same algorithm is run repeatedly starting with the more suboptimal solution (highest weighting) [53]. 

**Figure 2:**
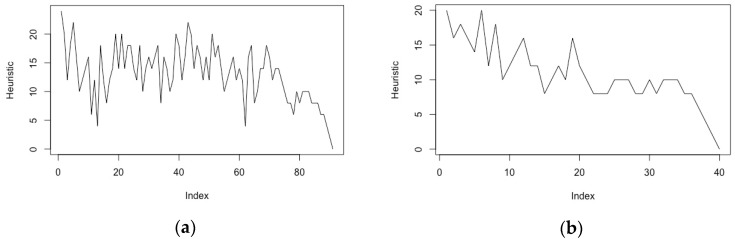
The impact of dynamic weighting on solution progression and backtracking. For the same configuration of the 8-puzzle (**a**) shows significant backtracking with a default heuristic function (A*), while in (**b**) WA* with fixed 0.575 weighting shows a more monotonic progression towards the goal state (as well as a faster computation).

We use a standard but complete A* implementation [45] (p. 75), which has been modified to incorporate dynamic weighting of its evaluation function, resulting in a weighted A* (WA*) implementation. In this version, dynamic weighting can take place from the onset of the search or be triggered once a certain percentage of the search space has been explored (for pre-set configurations such as the 8-puzzle). This version has supported both preliminary tests, which were dedicated to study search progression so as to determine how best to influence it (Figure 1 and Figure 2), and the actual BCI hybrid search experiments reported below.

Although heuristic search algorithms can be applied to a wide range of problems, the actual time dynamics of the search process differ significantly across problems, initial conditions, and search methods. A given search problem can be characterised by the shape of its search space, and the extent to which progression towards the solution is monotonic or requires extensive backtracking. 

For preliminary tests characterising solution progression, we have run various search problems from a database of previously resolved 8-puzzle configurations [54], which identify search problems (8-puzzle initial and goal configuration) in terms of number of solutions or solution length. Figure 1a shows the reduction in search space for various values of the WA* weighting coefficient, while Figure 1b illustrates the influence of intervention timing: the actual variation in search space for a fixed weighting coefficient depends on the stage of search progression at which dynamic weighting is applied.

In addition, some search problems place greater emphasis on the shape of the solution than on simply reaching the goal state. In this context, the impact of shifting to a non-admissible heuristic search would depend on the nature of the search progression itself (i.e., reducing the amount of backtracking or accelerating the monotonic progression towards the solution). It is thus necessary to confirm the ability of the bounded non-admissible search to improve search progression for different problem configurations.

Figure 2a shows the variation of the heuristic value during an 8-puzzle solution for a configuration known to have a 30+ move solution [54], with significant oscillations of the heuristic value indicative of extensive backtracking. Figure 2b shows the variation of the heuristic value for the same problem when shifting to a non-admissible search with a weighting value of 0.55 for the heuristic function.

### 2.2. The Integration Challenge

Integrating control of AI computations assumes a number of conditions for the implementation of the hybrid cognitive system framework. Firstly, the neural signal should be quantified, and its variation range should support a mapping onto defined parameters of the AI calculation. Such grounding can be found in the statistical correlations encountered in previous works, as well as the known magnitudes of signal variations above a baseline. For instance, when considering prefrontal asymmetry in a motivational framework, the situation should be differentiated between EEG and metabolic signals (fMRI, fNIRS), because the latter do not benefit from a fixed prefrontal asymmetry baseline, unlike the trait property of EEG prefrontal asymmetry [17]. 

Secondly, the signal should be controllable by subjects, implicitly or through an explicit cognitive strategy. The challenge here is to train subjects in developing cognitive strategies that are as specific to the task considered as possible and do not use confounding signals. For instance, prefrontal asymmetry is often influenced by valence in addition to approach, which explains that positively valenced cognitive strategies, such as personal autobiographic memories, can be successful in sustaining the BCI signal [27,55]. However, such cognitive strategies risk being distractive and are decorrelated from the observation of AI computation progress: this could constitute a case for NF training, which is generally reserved for clinical rather than user interface applications. The increase in NF performance, which is generally observed after a few training sessions, could support implicit, non-distracting cognitive strategies. Finally, the users should be responding to a real-time presentation of the progression of AI calculation so that their intervention is relevant in terms of influencing it. Several visualisation strategies will be introduced in the next sections. 

Volitional control should be implemented through BCI input supported by specific user training and cognitive strategies. Most literature using prefrontal asymmetry as an active BCI signal has implemented a NF paradigm, most probably because it sought inspiration from the significant literature on PFC asymmetry NF for clinical applications [27,56,57]. In this context, the user intervention can be best described as a motivational response targeting the current evolution of the AI computation. 

The integration process at the heart of BCI-controlled search relies on two main dimensions. The first one is the nature of the feedback signal used to convey a sense of search progression and direction: by giving the users a sense of how the search is progressing, it enables them to react accordingly on either time progression or, when available, the nature of the solution most likely to emerge. The second one is the temporal aspects of user intervention, which can be subdivided into timing and frequency of intervention. Timing refers to the time relation between user intervention and the overall duration of the AI computation: it is generally made possible by the extended nature of AI computations. When the progression of the solution can be conveyed meaningfully to the user, this may create the opportunity to guide the search process at various stages assuming again that the duration of the computation is significantly longer than the BCI epochs required for input. The repetition of interventions would then define a frequency of user interventions. 

In the next section, we review several options for implementing the above dimensions and how they can be combined to implement various BCI search paradigms. 

### 2.3. Intervention and Search Dynamics

It may seem a paradox to suggest a mechanism for interacting with an offline heuristic search algorithm, considering that there is no shortage of real-time variants of A*. However, there is a long history of repeatedly running heuristic search algorithms with modified heuristics to speed up the remainder of the computation, which was at the heart of various “anytime” variants of A* [48]. Making the search process responsive at specific progression intervals differs from the real-time heuristic search philosophy (e.g., RTA* [58]) in terms of heuristic value calculation (depth-bound lookahead versus goal state estimate in traditional search) and backtracking opportunities. For standard A* variants, the actual impact of overweighting the heuristic function towards non-admissibility varies greatly according to the stage of search progression at which it is applied and suggests that the options for intervention should take place over the early stage of the search progression, and this could be the case across a range of search problems (Figure 1b). 

This is the solution we have adopted in previous work, also owing to the response time of the fNIRS signal: it could however still be of interest even when using EEG-based input frontal asymmetry scores because of the signal dynamics and the need to stabilise it over the NF epoch. Moving towards some interruptible, anytime-like approach could bring the further advantage of buffering the BCI input rather than constraining the user input in terms of timing and dynamics. A particular implementation of the above consists of parameterising heuristic search from user profiling data prior to triggering AI computation. An essential condition for this parameterisation is the availability of a framework to unify search behaviour with user personality traits that would be readily accessible through BCI measurements. One such example would make use of prefrontal asymmetry under its electrical signal form (EEG), which has been shown to have trait properties [17], to characterise, in context, user disposition towards gain, reward or, risk. On the AI side, the above user dispositions can be interpreted as potential acceptance of various forms of suboptimal solutions. These dispositions could be translated into non-admissible variants of heuristic search trading optimality for speed. One more specific case would be the explicit use of a user’s risk propensity profile to be mapped onto an interpretation of risk in a heuristic search. Another core element of the system design is the timing and duration of BCI input. This design faces a number of constraints, from the user’s response time in assessing the progression of the AI computation to the onset of BCI signals and any difficulty in sustaining it. In addition, difficulties in controlling the magnitude of the BCI signal may be offset by repeated interventions throughout AI computation, subject to constraints on the intervention window for offline heuristic search. 

The difficulty in sustaining the BCI input signal is amply discussed in the NF literature and is one of the reasons for defining NF epochs of limited duration [59]. Moreover, even across defined NF training sessions, many recent papers have noted a drop in user BCI performance towards the latter epochs, which they have explicitly attributed to BCI fatigue. The difficulty for users to exert sustained control over specific brain regions activation is at the heart of BCI usability limitations. Leaving aside individual differences in ability, sometimes referred to as BCI illiteracy or non-responsiveness, which can be generic or specific to some BCI configurations, even the performance of a responsive subject tends to be inconsistent across trials. User task fatigue [60] has been particularly well documented during NF training involving a fixed sequence of epochs, with the performance of even good responders waning towards the last epochs of a training session. A practical consequence for BCI-controlled search would be to limit the number of user interventions in the course of any problem-solving session, as well as their duration.

### 2.4. Visualisation of Search Dynamics and User Response

There are a limited number of cases for which the problem being solved can be usefully visualised to give the user access to search progression towards a solution. Among the determinants making this possible are the spatial nature of the problem, the level of backtracking and monotonicity of solution construction, and the ability to derive a semantic interpretation from the search visualisation. One of the most straightforward examples is the use of heuristic search in path planning where the search progression can be visualised in real-time on the discretisation grid that supports the search process (see below, Figure 5). The overall progression can be made even more visible for complex obstacle densities and high probability of backtracking by highlighting those nodes of the grid that constitute the OPEN list. On the other hand, the tree-based visualisation of the search space of a puzzle (e.g., n-puzzle, Rubik’s cube…) is unlikely to offer sufficient insight to the user owing to the amount of information, difficulty of interpretation, and speed of search space expansion that generally exceeds human processing abilities in the absence of high-level detectable patterns. Such patterns are similar to those which would be encountered in board games but may only be visible to experienced players: in any case, we are not dealing here with adversarial heuristic search.

It is generally accepted that the feedback element of NF-based BCI helps the user in sustaining the activation of the target region of interest, even more so that the target is not under direct volitional control. This aspect has been discussed in the NF literature from multiple perspectives: the use and type of cognitive strategies, the classification of subjects into responders and non-responders, an ability to control the BCI signal that improves during training and the number of training sessions, and the positive impact of realistic feedback channels (e.g., games, virtual reality) over abstract visual indicators [61]. The ideal, long-term configuration, would be to use the visualisation of search progression itself as the NF signal: however, a major challenge to implement this approach would be to align the temporal aspects and sampling rates of the input BCI signal and the feedback signal. 

To a large extent, BCI-controlled search aims at influencing the exploration of the search space. It would then appear logical to present the users with some representation of the search space itself so that they would respond to the global shape of the search space from the initial to goal state. Assuming primarily a tree-based expansion, the traditional representation of heuristic search space is triangular (see for instance [45] (p. 152)). Moreover, the simple geometric shape and its natural interpretation in terms of ‘focussing’ the search to reduce the search space and expand more directly towards the goal can support a direct BCI feedback in the framework of a NF approach to BCI input, which has been shown to be appropriate to signals such as prefrontal asymmetry (Figure 4). 

**Figure 3:**
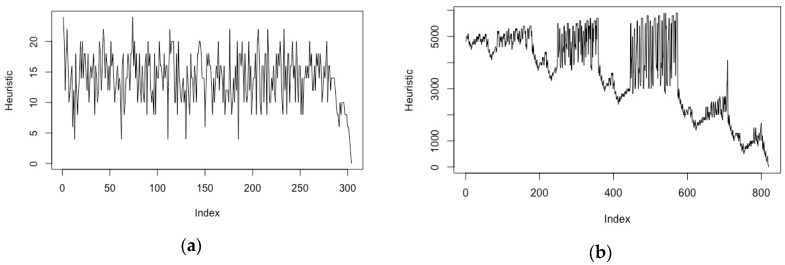
The variation of the heuristic function throughout the search process conditions the type of intervention (**a**) For the 8-puzzle problem, the heuristic function (Manhattan distance for misplaced tiles) oscillates significantly with search backtracking; (**b**) For a path planning problem, such as the one used in our preliminary experiments, there is an overall trend for the heuristic function (straight-line distance in arbitrary grid units) as the path progresses towards the goal node.

Although less immediately visual than the above abstract representations, search progression can also be represented through the time variation of the heuristic function values from the initial state to the solution state. It is only meaningful in terms of prompting user intervention when the heuristic shows a regular, ideally monotonic, trajectory towards the goal, such as on Figure 3b. On the other hand, heuristic value oscillations such as the one observed on Figure 3a for a classical 8-puzzle problem are not good candidates for such visualisation, because they do not converge until the very latest stages of the search. 

Influencing AI systems, as reported here, assumes a compatibility of timescales between AI computations, user perception of solution progression, and time constraints of NF input (response time, signal stability, and duration of an epoch). Despite progress made in AI techniques, typical search, planning, and optimisation problems still often require minutes of intensive computations to reach a result, as illustrated by standard benchmarks such as in the international planning competitions [62], where a cut-off time of 1800 s is introduced [63,64]. These timescales are much more representative of the target applications for our approach than examples such as the 8-puzzle used for proof-of-concept, which tend to be solvable in a few seconds. However, it should still be noted that the A* algorithm still today cannot scale up beyond simple problems [65], making non-admissible search and our overall approach still relevant. 

With NF epochs generally under 60 s, we would suggest that such timescales are close to optimality when it comes to designing human intervention, in particular for those problems exhibiting a heuristic progression profile such as the one of Figure 3b (which matches that of search-based planning (e.g., in [66])). 

## 3. Proof-of-Concept Experiments: BCI Control of Heuristic Search

In order to validate our motivational model, we carried out proof-of-concept experiments in which users could influence the course of heuristic search calculations using BCI input.

The motivational framework consists of trading solution optimality for speed of calculation: on the AI side, it is implemented through ε-bound heuristic search, and on the BCI side, the motivational element is captured through real-time variations of PFC asymmetry, measured using fNIRS NF. There is ample evidence that subjects can alter prefrontal asymmetry in real-time under a NF paradigm, using various cognitive strategies [27,56,67], some of which are clearly motivational (approach-based). We have in previous work successfully used prefrontal asymmetry as a BCI paradigm using both EEG with fMRI validation [68] and fNIRS [29,55]. 

Overall, the system comprises the AI component, which consists of a non-admissible A* implementation in the form of weighted A* (WA*) [47] operating on a standard heuristic search problem (8-puzzle or grid-based path planning), the fNIRS-based BCI interface that measures variations of prefrontal asymmetry from a baseline under a neurofeedback paradigm, a visualisation environment that supports the NF response and gives insight into the search space of WA*, and a mapping algorithm, which determines which variations of WA* weighting coefficients should be applied for the current variation of prefrontal asymmetry. 

The main objective of these experiments was to validate the motivational framework by showing that the BCI input can provide the necessary influence over the heuristic search computation in terms of information, bandwidth, and timing. Although this demonstrator does not yet implement all of the framework elements introduced in this paper (in particular, in terms of interaction timing and dynamics in relation to heuristic search progression), one important objective is to demonstrate some quantitative aspects of the mapping between BCI and heuristic search, namely that the magnitude of the user input can actually drive the computation towards various trade-offs between optimality and speed. We use one single integration paradigm, which is the precision–admissibility trade-off [45], also known as the optimality–time trade-off in cognitive research [50], where it is considered a motivational, approach-based implementation.

The common setting for the proof-of-concept experiments is based on a BCI NF paradigm, where active biofeedback is meant to support the user in controlling his/her prefrontal asymmetry. This is based on a large body of work that has demonstrated that prefrontal asymmetry could be controlled through NF across various types of BCI, electric (EEG) [68] or metabolic, in particular rt-fMRI [27]. In addition, previous research has established that the DLPFC, considered the main region involved in motivation-based PFC asymmetry [25,27], is readily accessible through fNIRS [69], including fNIRS NF [67,70]. Our NF protocol is primarily inspired by the rt-fMRI experiments on PFC asymmetry of [27], which helped us in defining epoch durations, time delays, magnitude of signal variation, and statistical validation. We have previously validated fNIRS PFC NF in a typical PFC asymmetry context dissociating approach from valence, which detected the expression of anger [29]. 

**Figure 4:**
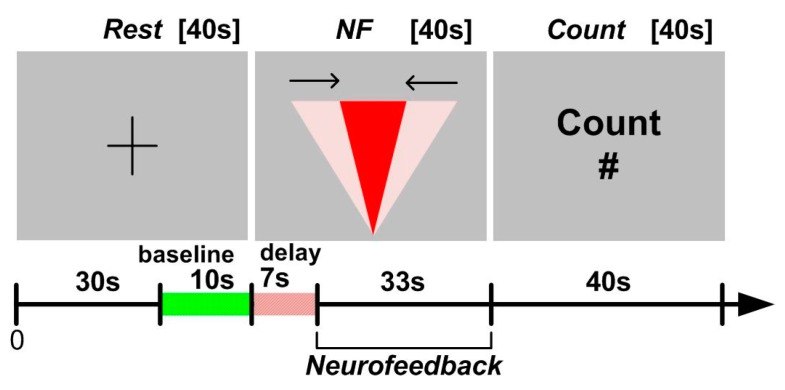
The neurofeedback (NF) protocol used for the 8-puzzle experiment. Note that the last 10s of a resting epoch are used to determine the prefrontal cortex (PFC) asymmetry baseline a priori to the NF epoch. The 7-s delay is introduced to take into account the onset of haemodynamic response in fNIRS. The NF epoch is followed by a non-motivational cognitive task facilitating the return to a new baseline [71].

The NF experiments are organised around specific sessions in which NF facilitates BCI input to influence AI computation: each session is composed of various blocks that enable baseline activity definition and BCI input itself. The details of block design and experiments can be found in [71] and are only briefly described here (see also Figure 4). The generic principle consists of having a single NF block compatible with the timing of fNIRS variations and serving as BCI input to control the AI computation. fNIRS being a metabolic method, there is no absolute baseline for PFC asymmetry like the one that exists in EEG measurements, imposing to recalculate a baseline asymmetry value before each NF block. Depending on the experiment, the baseline involves rest or an unrelated cognitive task (counting) not affecting PFC asymmetry. The asymmetry score computed during the baseline is used as reference and considered “zero asymmetry” regardless of its actual value. The last 10 s of the resting epoch (Figure 4) are used to measure that score with specific care taken not to induce variations of asymmetry. 

As with all NF installations, the feedback signal should be determined by the level of activation of the region of interest (here, the difference in activation between left and right PFC calculated by averaging oxy-haemoglobin (HbO) values over the four leftmost and four rightmost fNIRS channels, then subtracting the average right from the average left).

**Figure 5:**
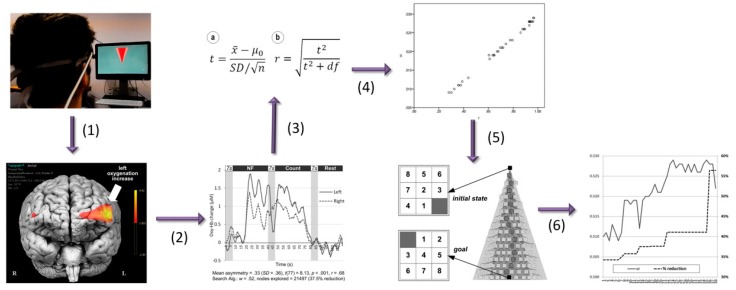
Brain–computer interfaces (BCI) interfacing to heuristic search on the 8-puzzle. The user’s motivational dimension is obtained through fNIRS measurement of PFC asymmetry (**1**). The level of change from the baseline, which is taken to measure approach, is determined with real-time statistical testing (**2**,**3**). It is mapped linearly onto the WA* weighting parameter using the effect size to determine the level of heuristic modification (**4**). The change in weighting parameter for WA* is applied during the search (**5**), which results in search space reduction and computation speed-up (**6**). Note the abstract representation of the search space as a two-dimensional (2D) beam (**1**), which serves as a visual feedback for fNIRS NF (adapted from [71]).

The first experiment explored BCI control over heuristic search for solutions to the 8-puzzle (Figure 5). The rationale for using a textbook example such as the 8-puzzle is that its complete solution set is fully accessible [54], which considerably simplifies the experimental design by selecting 8-puzzle configurations (starting state and goal state) whose properties are known. 

For instance, when applying heuristic weighting modifications during the search itself, it is possible to experiment with known solution lengths or configurations, admitting a large number of solutions to minimise the impact of dynamic modifications. Because the range of solutions and impact of ε-admissibility is documented, the mapping of BCI input to heuristic search is also easier to describe and experiment with. 

The mechanism by which a feedback signal is generated from the detection of BCI input is generally referred to as mapping and plays an important role in NF design (Figure 5(4)). Here, the starting point to determine the best mapping functions is to look at the outcome of non-admissible search experiments. These determine the range of heuristic function modifications that have the most significant effect in terms of performance–admissibility trade-off. Previous literature on non-admissible search [47,48] has established a number of principles, such as the fact that the main impact of non-admissibility is to reduce the size of the search space or that significant effects could be observed for even minor modifications of the heuristic weighting. In our experiments, the BCI signal (level of asymmetry compared to the baseline) is mapped linearly onto an abstract symbology for the search space taking the form of a two-dimensional (2D) beam whose width represents the variable to be minimised. We have based the mapping on the statistical significance of fNIRS signal variation with respect to the baseline using real-time t-tests and associated effect size (Figure 5(3)). The post-hoc validation of each NF epoch has been confirmed using resampling methods, in particular bootstrapping [72].

The intervention model for the 8-puzzle was to request a NF intervention soon after the start of the search process, resulting in the heuristic weighting being altered after 0–25% of the search space had been explored (this value being derived from the known solution configuration, see Figure 1).

In the case of the 8-puzzle, the main impact was on search space reduction, measured through a reduction in the number of nodes expanded [48] and consistent with our preliminary tests of non-admissible search (Figure 5(6)). It is worth noting that the optimality of the solution was actually often preserved, meaning that the users were actually successful in speeding up the AI computation without compromising solution quality.

The variations in prefrontal asymmetry across subjects resulted in differentiated effects on heuristic weighting and associated search space reduction, compatible with the intended quantified use. However, there was not enough data in our single-trial experiments to assess intrasubject variations and validate how a single subject could fine-tune the behaviour of a given search progress. This raises the issue of the controllability of the magnitude effect, which should be the object of further experiments but could also be mitigated through multiple interventions during a given AI computation.

**Figure 6:**
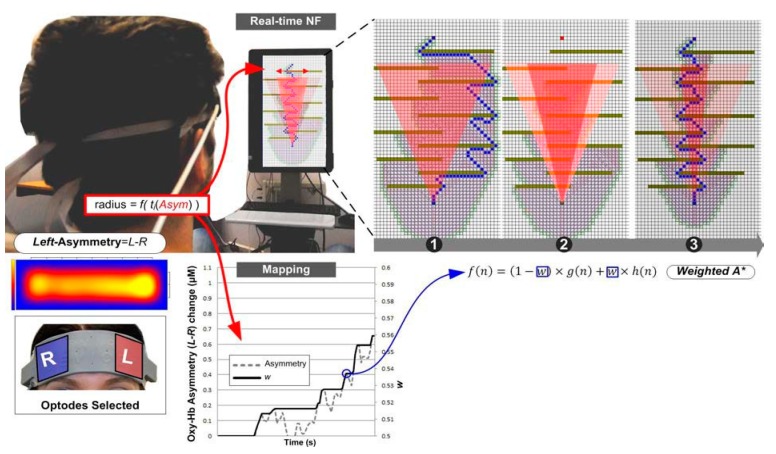
BCI interfacing to heuristic search on a path planning problem. The motivational dimension is acquired through fNIRS-based prefrontal cortex asymmetry. In this experiment, the heuristic function can be repeatedly modified as the search progresses, also taking advantage of the more visual feedback provided by path progression. Because of the multiple updates, in this experiment the weighting factor has only been allowed to increase through time to explore search speed-up. Note the change in the qualitative nature of the solution (path geometry) from solution (1) to solution in (3). From [71].

A second set of experiments was staged using grid-based path planning as a heuristic search problem (Figure 6). The rationale for this second test case was that the search space could be visualised in real time as the search progressed so that the visual feedback sent to the user about search progression was no longer metaphorical. However, to avoid potential uncanny effects due to the shape of the node frontiers progression (which with grid-based path planning also depends on obstacle density and environment layout), the display superimposed the same triangular shape over the node progression to be used as the NF channel. In this second experiment, the search space is comparatively smaller, and the reduction in search space is less dramatic than with the 8-puzzle. However, non-admissible search produces qualitative, as well as quantitative modifications of the solution path, which can be readily observed on the chosen obstacle configuration: the solution path under user intervention is more straightforward and travels through the centre of the environment.

Interestingly, the success rates did not differ significantly from the 8-puzzle experiments, suggesting that a better visibility of the search space progression did not improve subjects’ performance in that instance. However, this might depend on the actual obstacle density and layout, as the actual shape of the front node progression and associated backtracking might actually be distracting to users. 

The users’ perception of the task can be analysed through their narrative feedback on the cognitive strategies they used to increase prefrontal asymmetry. Several users reported strategies compatible with approach and result anticipation such as imagining running in a virtual race or encouraging the progression of the search as one would encourage a racer. Prior to the experiment, subjects were explained the goal of AI computation and the NF setting, although we refrained from suggesting explicit cognitive strategies. However, a few others mentioned the recollection of positive autobiographic memories, which is known to also induce left prefrontal asymmetry because of the interplay between valence and approach in appetitive stimuli or recollections, as also reported by Zotev et al. [27] in their fMRI prefrontal asymmetry NF experiments. 

In these experiments, NF success is defined for each subject as having at least half of successful blocks during a NF trial [72]: this high-level measure is meant to give an indication on the usability of the interactive system.

It is interesting to compare current success scores to two other previous fNIRS experiments also involving PFC asymmetry in two different affective contexts (engagement (Aranyi et al. [55]) and anger (Aranyi et al. [29])). All these experiments have in common a minimal level of user training which tends to be the same across experiments: the calculation of PFC asymmetry from haemodynamic data is similar, based on the same optodes and the same formula. Previous affective BCI experiments resulted in success scores of 73% [55] and 70% [29]. Our new 8-puzzle experiment achieved a similar score of 73%, suggesting that significant NF success is possible in the absence of a clear affective context, with a motivational-based approach for which there is no priming from the application or visual environment. Paradoxically, the increased visual realism in the path planning setting did not result in higher success scores, despite the reported positive impact of visual realism on NF [61]. Based on debriefing and narrative feedback from the subjects, the lower success scores observed for path planning (57%) were attributable to the extra cognitive load induced by the visual complexity. Another potential explanation is that in the path planning experiment, the baseline was determined during the counting epoch rather than during a post-counting resting epoch; although counting is considered a neutral task for prefrontal asymmetry, it could in some cases affect it via mental workload for some subjects [73], thereby introducing a ceiling effect in PFC asymmetry variation with subsequent impact on success scores. 

Another important point to consider when analysing performance is that we are using NF as an interaction paradigm rather than as a therapeutic approach. Of NF, we only retain the hypothesis according to which the presence of the feedback signal helps the user activate brain regions not directly accessible to volitional control. Unlike NF therapeutic systems we do not include multiple training sessions, which are used to induce long-term behavioural changes (mediated by neural plasticity) and are generally associated with an improvement in the ability to control the NF signal throughout training. This induces an inherent limitation in our approach, which is that overall subject performance will generally be lower in the absence of multiple training sessions. The minimal training provided to our subjects can be counted in minutes, whilst it is generally considered that several hours (up to 40, [74]) through repeated sessions are required for subjects to be confident with NF control. In practice, subjects were allowed between one (path planning) and three (8-puzzle) blocks for training, which, considering the maximum block length of 120 s, can safely be considered as mere familiarisation rather than training across multiple sessions. 

One objective of the proof-of-concept experiments was to demonstrate the users’ ability to control prefrontal asymmetry in a generic motivational context related to the expectation of a computation result, this expectation taking the shape of a trade-off between quality and performance. This objective is highly specific to the possibility to control AI systems and differs from previous BCI use of PFC asymmetry, which has been primarily involved with affective BCI [29,55]. This difference arises from the generic motivational model associated with PFC asymmetry, which can be connected both to reward expectation and to appetitive stimuli, the latter going as far on the affective spectrum as to constitute a high-level dimensional aspect for empathy. In all our previous affective BCI work, a strong context, both prior to the NF trials and during trials themselves, may have facilitated user control. For instance, in eliciting anger against a virtual agent, subjects have been shown short videos evidencing the bad character of the agent [29]; in eliciting empathy or support, they have followed a narrative showing the character in trouble [68]. No such context is available when considering the control of algorithmic AI progression: moreover, as we are using abstract benchmark examples that do not even correspond to popular board games, it appears essential to assess how users can operate in the absence of a direct sense of reward expectation, other than the one conveyed to them as part of the experiment brief. 

## 4. Conclusions and Further Work

We have introduced a framework inspired by human augmentation for the control of autonomous AI systems, which opens the way to the development of new interaction technologies dedicated to human–AI cooperation. This framework departs from previous research in that it seeks to adapt to the imbalance between high-performance autonomous AI systems and users’ information processing abilities and response times, which require the latter to operate at specific levels of abstraction. The description of this framework has uncovered a number of important design issues, amongst which are the synchronisation of BCI input and AI computations and the leverage effect that basic AI mechanisms such as search will have on global computation. The former aspect will prescribe under which conditions BCI-input delays provided by metabolic methods such as fNIRS can be accommodated or whether the system should resort to EEG measurements of motivational parameters. Our proof-of-concept experiments have only examined traditional search problems, without addressing the potential leverage that the search will bring onto higher-level AI computations. One candidate technique to further this aspect of the research would be to examine heuristic search planning systems [43]. One notable element is that the heuristic function in some heuristic search planning applications tends to follow a trend similar to that of Figure 3b [66]. 

Throughout our early work, we have opted for single NF sessions with a limited number of epochs, supported by cognitive strategies. Although we have not been prescriptive about the type of cognitive strategies to be used, we have introduced subjects to the concept of cognitive strategy, as “thought contents” that would lead to best performance in the NF task. The role played by cognitive strategies can be explained in part by the fact that these experiments implemented single-session NF: although the actual requirement for cognitive strategies in NF has been debated [75], repeated training sessions may be required for subjects to perform without the help of a cognitive strategy. 

It now appears that too much emphasis on cognitive strategies may actually distract users from the observation of AI computation progress, which should be the primary driver of their BCI input. In the future, this could be addressed through two complementary directions. One would consist of a more comprehensive use of the AI computation progress as a visualisation feedback channel to support BCI input: however, this approach would require non-trivial temporal alignment between AI progression visualisation and the NF interface, which could require buffering, warping, or predictive features to be incorporated. Another direction is to accept the need for extensive NF training to support users’ performance: typical training times reported range from a few hours to up to 40 h [74]. 

Even restricting ourselves to a motivational model, it is not always possible to distinguish whether variations in prefrontal asymmetry should be interpreted in terms of approach [17] or in terms of risk taking [52]. This is part of a broader issue, well described in prefrontal asymmetry research, known as the balance of activity variation across each hemisphere that accounts for the observed increase in left asymmetry (because left asymmetry is the target in our experiments). During our previous experiments on PFC asymmetry [29,55,68], most of the increase in prefrontal asymmetry could be attributed to a proportionally greater increase in left-side rather than right-side activity. It has proven elusive to observe a selective decrease of right PFC activity, even a relative one, as a mechanism for left asymmetry, including in the experiments upon which we are commenting here, suggesting that increased risk taking cannot be considered as a primary mechanism. However, recent EEG NF work has evidenced such selective decrease in right prefrontal activity [57]. If this latter effect could be reproduced in a hybrid cognitive scenario, it could open the way to a risk–acceptance paradigm, as discussed above. A successful implementation of a risk paradigm would have significant interest in terms of AI applications, provided it ensures that users have an appropriate perception of alternative solutions in terms of risks.
